# Animal Venoms as Potential Source of Anticonvulsants

**DOI:** 10.12688/f1000research.147027.1

**Published:** 2024-03-27

**Authors:** Syafiq Asnawi Zainal Abidin, Anthony Kin Yip Liew, Iekhsan Othman, Farooq Shaikh

**Affiliations:** 1 Monash University Malaysia, Jeffrey Cheah School of Medicine and Health Sciences, Monash University, Bandar Sunway, Selangor, 47500, Malaysia; 2 School of Dentistry and Medical Sciences, Charles Sturt University, Orange, New South Wales, 2800, Australia

**Keywords:** Epilepsy, Anticonvulsant, Animal Venom, Bioactive Compounds, GABA, NMDA

## Abstract

Epilepsy affects millions of people worldwide, and there is an urgent need to develop safe and effective therapeutic agents. Animal venoms contain diverse bioactive compounds like proteins, peptides, and small molecules, which may possess medicinal properties against epilepsy. In recent years, research has shown that venoms from various organisms such as spiders, ants, bees, wasps, and conus snails have anticonvulsant and antiepileptic effects by targeting specific receptors and ion channels. This review underscores the significance of purified proteins and toxins from these sources as potential therapeutic agents for epilepsy. In conclusion, this review emphasizes the valuable role of animal venoms as a natural resource for further exploration in epilepsy treatment research.

## 1. Introduction

Epilepsy is a global epidemic that affects more than 50 million people worldwide, making it one of the most common neurological diseases. ^1^ Almost 80% of epileptic patients come from low- and middle-income countries. ^1^ The Task Force of the International League Against Epilepsy (ILAE) defined epileptic seizure as a transient occurrence of symptoms caused by abnormally excessive brain neuronal activity. Epilepsy is a brain disorder prone to epileptic seizures and consequences from cognitive, neurobiological, psychological, and social aspects. ^2^ There are several types of epileptic seizures, depending on the symptoms. One-third of the epileptic seizures occurred as generalized seizures, whereas two-thirds began as focal seizures. Generalized seizures can be classified into six groups: tonic-clonic, myoclonic, atonic, tonic, clonic, and absence seizures. ^3^ All these symptoms happen without warning and involve loss of consciousness on the patient. Tonic seizures are characterized by constant muscle contraction, while tremors in the limbs indicate clonic seizures. Tonic-clonic seizures have symptoms that combined both tonic and clonic seizures symptoms. The contraction of limb muscles is followed by limb extension and tremors with back arching for around ten to thirty seconds.

Epileptogenesis is the gradual development of epilepsy in a normal brain. ^4^ The causes of epilepsy can be identified as structural or genetics. Structural causes of epilepsy include brain tumors, brain trauma, and other neurodegenerative diseases. Brain areas prone to trauma causing epileptogenesis include structures of the temporal lobe such as the amygdala, hippocampus, and piriform cortex. ^5^ Brain electrical activity is usually non-synchronous in normal humans, while patients suffering from epileptic seizures will have excessive synchronized firing of neuronal firing, which causes a paroxysmal depolarizing shift. Neuronal biochemical receptors play an essential role in epileptogenesis, primarily glutamate receptors (ionotropic and metabotropic). When the receptors are activated, Ca2+ ions will increase within the area of the cells. Therefore, glutamate is seen as one of the most critical molecules in epileptogenesis as it causes excitotoxicity, i.e., neurons are severely depolarised. ^4^

Epilepsy treatment may include drugs, surgery, and even diet changes. The standard first-line therapy for patients newly diagnosed with epilepsy is usually by antiepileptic drugs (AEDs) such as phenobarbital, phenytoin, carbamazepine, or valproic acid. Second line medications such as oxcarbazepine, gabapentin, topiramate, felbamate, and lamotrigine are also applied in the treatment. ^6^ No single AED is proved to be more effective than another, with all drugs producing some side effects depending on the individual treated. These drugs used have a 70% to 80% success rate in treating epilepsy, while the remaining 20% to 30% still suffer from seizures or more significant adverse effects. ^7^

Since epilepsy is caused by the malfunction of ion channels, Gamma-aminobutyric acid (GABA)-ergic neuron inhibition, and excess glutamate release, most AED will target these specific pathways. ^8^ Drugs that target ion channels include carbamazepine, oxcarbazepine, phenytoin, and lamotrigine, which block sodium channels. ^9^ Gabapentin binds on volt-age-dependent calcium channel proteins in rat models and is believed to have GABAergic effects. GABA-mediated inhibition is vital as the binding of GABA to their receptors leads to an influx of negatively charged chloride ions (Cl-) which in turn causes cell hyperpolarisation. Barbiturates and benzodiazepines treat epilepsy by targeting the GABAergic transmission and increasing chloride ion influx. ^10^ Glutamate, which is a primary excitatory neurotransmitter were also believed to play a part in epilepto-genesis. Application of antagonists to the glutamate receptor such as N-methyl-D-aspartate (NMDA) demonstrated anticonvulsant effects in animal models. Valproate, felbamate, and topiramate are NMDA receptor antagonists which are currently used. However, these drugs are known to present several undesirable side effects. ^8^

## 2. Animal venom as a potential source of anticonvulsants

The earth’s flora and fauna are an important reservoir of naturally derived therapeutic agents. Since ancient times, humans have utilized these resources to treat diseases and improving wellbeing. One significant source is the venom and toxins from venomous and poisonous animals. The venom consists of proteins, enzymes, inorganic ions, and nucleotides, which can induce a plethora of effects, including necrosis, coagulation, haemorrhagic, and neurotoxicity depending on the type of venom. ^11^ Targeted sites of venom are specific and diverse due to the different constitutions and may target receptors and channels such as neuro-junctions and ion channels. ^12^ Animal venom has been regarded as an essential source of potential therapeutic agents in human diseases such as neurodegenerative diseases, cardiovascular diseases, autoimmune diseases, and cancer. ^13^^–^^16^ In this literature review, the different types of venom used in treating epilepsy is discussed.

### 2.1 Spider venom ( Table 1)

Neuroprotective potential of various venom fractions and components from venomous spider species has been identified ( Table 1). Arylamine-enriched fraction isolated from the venom of funnel-web grass spider *Agelenopsis aperta* demonstrated significant anticonvulsant properties by antagonizing and blocking NMDA receptor and dihydropyridine (DHP) calcium channels. ^17^ Overexcitation of NMDA receptors and DHP calcium channels were found to be responsible for the pathophysiology of epilepsy. ^18^^,^^19^ In the study by Jackson and Parks, ^17^ arylamine-enriched venom fraction was administered both intravenously (i.v.) on male Sprague-Dawley rats induced with epilepsy using kainic acid (KA), picrotoxin (PIC), and bicuculline (BIC). Subjects administered with KA when paired with the venom fractions demonstrated less to nonconvulsive behavior until 70 minutes. Symptoms of seizures subsided after 4 hours with no deaths observed in the 10 subjects. Subjects administered with BIC and PIC paired with the venom fraction (i.v.) also shows closely similar observations and seem mildly sedated at the same time responsive to external stimuli.

**Table 1.** Anticonvulsive proteins/peptides from spider venom and the mechanism of action.

Species	Venom Component	Mechanism	Reference
**Spiders**			
*Agelenopsis aperta*	AG2	NMDA receptor antagonists/blocks dihydropyridine (DHP) sensitive calcium channels	^17^
*Parawixia bistrata*	Parawixin 2/FrPBAII	Blocks GABAergic transporters, interferes GABA uptake	^24^^,^^27^
*Parawixia bistrata*	Parawixin 10	Increases glutamate uptake	^25^
*Scaptocosa rapto-ria*	SrTx1	Blocks GABAergic transporters, interferes GABA uptake	^23^
*Parawixia bistriata*	Peptide fraction (RT10)	In-vitro neuroprotective effect against excitotoxicity	^26^

NMDA = N-methyl-D-aspartate; GABA = gamma-aminobutyric acid.

Active fraction (PbTx1.2.3) from Brazillian orb-weaver spiders *Parawixia bistrata* demonstrated potential anticonvulsive activity in synaptosomes model from rat cerebral cortex. ^20^ Glutamate and gamma-Amnobutyric acid (GABA) uptake balance is crucial in maintaining normal brain function. Functioning as the primary excitatory neurotransmitter in the brain, the increase of glutamate activity is critical in epileptogenesis. ^21^ In contrast, GABA counterbalances neuronal excitation by acting as the primary inhibitory neurotransmitter. Hence, changes to GABA balance in the brain may trigger seizures. ^22^ PbTx1.2.3 was demonstrated to increase glutamate uptake while inhibiting GABA uptake in cortical synaptosomes in a dose-dependent manner. Drugs that stimulate GABA concentration are potent anticonvulsants such as vigabatrin and tiagabine. ^22^ Moreover, PbTx1.2.3 successfully protected neurons in the rat retinal glaucoma model from excitotoxic death. ^20^ Therefore, PbTx1.2.3 may serve as a template for potential anticonvulsant and neuroprotection drug development.

FrPbAII is another compound found in *P. bistrata* venom, which was studied for anticonvulsive and anxiolytic properties. Bicuculline was injected into *Area-tempestas* of male Wistar rats to induce seizures and treated with FrPbAII. The toxin component significantly inhibited seizures elicited by bicuculline. In addition, FrPbAII was demonstrated to block GABAergic transporters that caused epilepsy. ^23^ Animal behavior study using male Wistar rats also showed anticonvulsive properties of FrPbAII. Rats were induced with seizures using pentylenetetrazol and were treated with diazepam, nipecotic acid, and FrPbAII. All subjects treated with diazepam, nipecotic acid, and FrPbAII showed anticonvulsive properties with reduced immobility, increased exploratory walking, rearing, and head-dips than saline control pentylenetetrazole (PTZ)-induced seizure subjects. ^24^

Parawixin-10 is another polyamine found in *P. bistrata* venom which elicits anticonvulsive properties. Parawixin-10 was administered via i.c.v. injection using a cannula and kainic acid (KA), NMDA, and PTZ to induce seizures. Parawixin-10 demonstrated anticonvulsive properties with the lowest dose required for KA compared to NMDA and PTZ indicating that the mode of action for Parawixin-10 is through the inhibition of excitatory transmission of non-NMDA receptors. Neurochemistry data from the study also showed an increase in L-glutamate and glycine uptake, wherein elevated levels of both compounds are responsible for epilepsy. However, the important mode of action for Parawixin-10 has yet to be pinpointed and thus requires further investigation and studies. ^25^ Additional study on *P. bistrata* venom fraction, RT10, which comprised of Parawixins 1 and 2, demonstrated neuroprotective effects on neuron-glia primary culture against high L-glutamate concentrations. ^26^

### 2.2 Scorpion venom

Several subtypes of α and β neurotoxins have been described from the venom of Asian scorpion *Buthus martensi* Karsch (BmK) ( Table 2). These neurotoxins were found to modulate voltage-gated sodium channels (VGSCs). ^28^^,^^29^ Antinociceptive effects from BmK venom were demonstrated by the activity of BmK I (α-like neurotoxin) and BmK IT2 (β-like neurotoxins). ^30^^,^^31^ A study by Zhao *, et al.* ^32^ in PTZ and pilocarpine-induced epilepsy rats showed significant anticonvulsant activity from the administration of BmK IT2. The effects of BmK IT2 were attributed to its ability to modulate sodium channels in the hippocampus. A further study with a different toxin from BmK, Bmk-AS, demonstrated similar antiepileptic activities in PTZ and pilocarpine-induced epilepsy in the rat model. ^33^ In another study, a novel peptide synthesised and derived from the Malaysian forest scorpion venom, *Hetermetrus spinifer*, was shown to reduce inflammation in PTZ-induced epilepsy in-vivo. ^34^ The 25 amino acid peptide named HsTx2 alleviate epilepsy progression by targeting the circ_0001293/miR-8114/TGF- β2 axis, which is responsible for inflammation response in astrocytes. ^34^

**Table 2.** Anticonvulsive proteins/peptides from scorpion venom and the mechanism of action.

Species	Venom Component	Mechanism	Reference
**Scorpions**			
***Buthus martensi* Karsch**	BmK IT2	Interferes with voltage gated sodium ion channels	^32^
***Buthus martensi Karsch***	Bmk AS	Interferes with voltage gated sodium ion channels	^33^
***Heterometrus spinifer***	HsTx2	Regulation of inflammatory responses through the circ_0001293/miR-8114/TGF-β2 axis	^34^

### 2.3 Bee and Wasp venom

Bee and wasp venom is rich in compounds that act on various biological systems, especially in the nervous system. These compounds include peptides, amines, enzymes, and amino acids ( Table 3). ^35^ Both peptides and acyl polyamines have been studied to treat many neurology-related diseases. A single study on mellitin, a major component of the venom from Apis mellifera (honey bee), demonstrated anticonvulsant effects in bicuculine-induced seizures in rats. ^36^ Mellitin could potentially induced its neuroprotective activities via activation of phospholipase A _2_ and consequently modulating the GABAergic and glutamergic systems. ^36^ Denatured crude venom from *Polybia occidentalis* (Yellow-banded Polybia wasp) identified dose-dependent effectiveness in treating KA-induced seizures rat model. This finding suggests that venom-induced anticonvulsive activities by antagonizing NMDA-glu receptors. Additional results include *in-vivo* anticonvulsive effects in rats with PIC- and BIC-induced seizures. ^37^ Recent study on a venom peptide isolated from *Polybia occidentalis*, occidentalin-1202, pinpointed anti-seizure effects by blocking glutamate and kainic acid interaction with the kainite receptor. ^38^ A different study on the venom of *Polybia ignobilis* (Brazilian social wasp) demonstrated a similar outcome by preventing seizures induced by KA, PIC, and BIC. ^39^

**Table 3.** Anticonvulsive proteins/peptides from wasp and bee venom and the mechanism of action.

Species	Venom Component	Mechanism	Reference
**Wasps**			
***Polybia occidnetalis***	Denatured crude venom	NMDA receptor antagonists/increases glutamate uptake	^37^
***Polybia ignobilis***	Denatured crude venom	Interferes with glutamate and GABA receptors	^39^
***Polybia paulista***	Peptide fractions	GABA receptors and calcium ion channel interference	^40^
***Agelaia vicina***	Crude boiled venom	Interferes with GABA and glutamate uptake	^42^
***Philanthus triangulum* F.**	β-PTx/δ-PTx	Inhibits glutamate uptake	^43^
***Polybia paulista***	Ppnp7/Neuropolybin	Neuroprotective effects against tonic-clonic seizures in PTZ-induced rats	^41^
***Polybia occidentalis***	Occidentalin-1202	Inhibit glutamate uptake through blockage of kainate receptors	^45^
***Chartergellus communis***	Chartergellus-CP1	Postulated to block calcium channels	^44^
**Bees**			
***Apis meliffera***	Melittin	Increases GABA transmission, modulation of glutamatergic neurotransmission	^36^

NMDA = N-methyl-D-aspartate; GABA = gamma-aminobutyric acid.

Low molecular weight fractions of the venom from *Polybia paulista* (Neotropical social wasp) exhibited anticonvulsant activities in 60% of animals (Wistar rats) induced with PTZ. ^40^ Compared with diazepam treatment, both offer protection from seizures, but the venom demonstrated fewer sedation effects. ^40^ Additionally, peptides isolated from *Polybia paulista*, Ppnp7 and neuropolybin, demonstrated tonic-clonic seizure protection in 80% of PTZ-induced rats. ^41^ *Agelaia vicina* venom showed inhibition of GABA and glutamate uptake responsible during epileptogenesis. The venom targeted enzymes accountable for the regulation of ion flow, thus disrupting ion concentration gradient. Additionally, free amino acids were found to inhibit GABA and glutamate uptake. ^42^ *Philanthus triangulum* F. wasp venom contains components such as β-PTX and δ-PTX, which show effects on animal glutamatergic neuro-junctions. These components were found to inhibit the uptake of glutamate in the neuromuscular synapse on locusts. A study showed a significant decrease in glutamate uptake on hippocampal slices of rats incubated with δ-PTX (74% decrease) and a less significant reduction with incubation in β-PTX (18%). Based on the findings, the venom from *Philanthus triangulum* F. wasps demonstrated potentials as a source of anticonvulsant agents due to its interference with glutamate uptake, a critical factor in epileptogenesis. ^43^ Besides PTZ-induced seizures, a treatment using wasp venom peptide from *Chartegellus communis* (Social wasp), Chartergellus-CP1, showed neuroprotective effects in pilocarpine-induced epilepsy. ^44^ In the study, electroencephalographic abnormalities that are associated with pilocarpine-induced seizures were significantly reduced upon administration of Charthergellus-CP1. ^44^

### 2.4 Ant venom

Ant venom contains an arsenal of molecules that ranges from proteins, peptides, free amino acids, alkaloids, formic acid, and several other organic molecules. ^46^ Like other venomous organisms, the venom has been a subject of interest to uncover its properties and therapeutic potential, including as an anticonvulsive agent ( Table 4). The venom of *Dinoponera quadriceps* (South American giant ant) has been studied for anticonvulsant effects. An initial study by Noga *, et al.* ^47^ demonstrated the pro-and anticonvulsant effects of the crude venom *in-vivo.* Injection of crude venom (DqTX) in the lateral ventricle region of mice brain showed procursive behavior and tonic-clonic seizures.

**Table 4.** Anticonvulsive protein/peptides from ant venom and the mechanism of action.

Species	Venom Component	Mechanism	Reference
**Ants**			
***Dinoponera quadriceps***	Crude venom DqV	GABA receptor interference	^48^
***Dinoponera quadriceps***	DqTx 1 – DqTx 6	GABA receptor interference	^49^
***Dinoponera quadriceps***	M-PONTX-Dq3a, M-PONTX-Dq3b and M-PONTX-Dq3c	Decreased production of reactive oxygen and nitrogen species	^38^

GABA = gamma-aminobutyric acid.

Interestingly, the effects were negated via pre-administration of the same but denatured venom (AbDq), hence, protected the animals against seizures and death. ^47^ Similar results were noted in another study by Lopes *, et al.* ^48^ Different administration routes of *D. quadriceps* venom in mice seizure models were responsible for neuroprotective or neurotoxic effects. Intraperitoneal administration of the venom demonstrated increased latency to seizure with an increased survival rate in the PTZ-induced seizure model. In contrast, endovascular treatment-induced neurotoxic effects by reducing the seizure latency time. In another study utilising PTZ-induced seizure model, three different peptides from Dinoponera quadriceps (M-PONTX-Dq3a, M-PONTX-Dq3b, and M-PONTX, Dq3c) also showed anticonvulsant properties by reducing neuroinflammation caused by reactive oxygen species. ^38^

In a subsequent study by Noga *, et al.,* ^49^ the anticonvulsant effects from the fractions of *D. quadriceps* crude venom were investigated. The crude venom was fractionated using high-performance liquid chromatography (HPLC) and yielded six fractions labelled DqTX1 – DqTX6. The fractions were administered intracerebroventricularly in BIC-induced seizure rats, and the anticonvulsant effects were determined. DqTX-6 showed significant neuroprotection with 62.5% protection from seizures and 100% protection from death. Since BIC induces seizures by interfering with GABA receptors, the mode of action for the DqTX-6 fraction is most likely to be GABAergic. ^49^

### 2.5 Conus snail venom

Conopeptides from the venom of marine cone snails belonging to the genus *Conus* have been identified with potentials as antinociceptive, antiepileptic, neuroprotective, and cardioprotective agents. ^50^ There are at least four peptide families with neuroprotective and cardioprotective activities from *Conus* snail venom, namely, conantokins, omega-conotoxin (ω-Conotoxin), mu-conotoxin (μ-Conotoxin), and kappa-conotoxin (κ-Conotoxin) ( Table 5). ^51^ Conantokins are of particular interest due to their potential in treating epilepsy. Seven types of conantokin peptides were identified, and all were described as NMDA receptor antagonists. ^52^

**Table 5.** Anticonvulsive proteins/peptides from cone snail venom and the mechanism of action.

Species	Venom Component	Mechanism	Reference
**Cone snails**			
***Conus geographus***	Con-G	NMDA receptor blocker	^57^
***Conus lynceus***	Con-L	NMDA receptor blocker	^59^
***Conus radiatus***	Con-R	NMDA receptor blocker	^58^
***Conus magus***	Ziconotide	NMDA receptor blocker, N-type calcium channel blocker	^60^

NMDA = N-methyl-D-aspartate.

Conantokin-G (Con-G) is a well-studied conantokin from the venom of Geography cone snail *Conus geographus.* It was advanced into clinical trial phase 1 to treat intractable epilepsy and potential neuro-protectant in ischemic stroke. ^53^ The peptide demonstrated significant antiepileptic activities in animal models of epilepsy, such as maximal electroshock, PTZ induced, and Frings audiogenic seizures. ^54^^–^^56^ Anticonvulsive mode of Con-G was via the dose-dependent inhibition of NMDA responses. ^57^ Besides Con-G, Conantokin-R (Con-R) from *Conus radiatus* Conantokin-L (Con-L) from *Conus lynceus* showed significant anticonvulsant effects when administered intracerebroventricularly to subjects induced with Frings audiogenic seizures. ^58^^,^^59^ Ziconotide, a synthetic derivative of ω-Conotoxin from *Conus magus*, demonstrated anticonvulsant and anxiolytic effects by potentially reducing extracellular concentrations of glutamate via N-type calcium channel inhibition. ^60^

## 3. Future directions

The potential of venom as a source of anticonvulsants warrants further exploration and investigation, extending beyond its current focus to encompass other venomous animals which could potentially include snakes. Snake venom is a complex mixture of proteins, peptides, and small molecules that serve as a defence mechanism and aid digestion for snakes towards their prey. Despite its toxicity, snake venom can be an essential source of therapeutic compounds, as demonstrated by its potential in treating cardiovascular disease and as an anti-microbial, anti-viral, anti-fungal, and anti-cancer agent. ^61^ To date, there is only a few studies the anticonvulsant potential of snake venom via *Ophiophagus hannah* (king cobra) and *Micrurus mipartus* (Costa Rican coral snake). Saha *, et al.* ^62^ isolated a non-protein toxin called KC-MMTx from *O. hannah* crude venom and demonstrated its neuroprotective activity in animal models of epilepsy. KC-MMTx presented significant protection against seizures induced by strychnine, PTZ, and yohimbine, and its potential in interfering with the GABAergic and glycine receptors was touted. Remarkably, toxins from Costa Rican coral snake (MmTX1 and MmTX2) were found to specifically target GABA receptors and significantly improve its sensitivity via allosteric binding. ^63^ Moreover, the ability of snake venom toxins, particularly three-finger toxins (3FTXs), to bind with nicotinic acetylcholine receptors (nAChR) presents another promising avenue in this field. This is noteworthy because the overactivation of nAChR is frequently associated with tonic-clonic convulsions and epilepsy in mammals. ^64^^–^^67^ In addition to snake venoms, venoms from sea anemone could also be another important source of therapeutic proteins and peptides as anticonvulsants. ^68^ Several sea anemone toxins extracted from *Stichodactyla heliantus*, *Actinia equina*, *Anemonia eryhtrae*, *Anemonia sulcata*, *Bunodosoma caissarum, Bunodosoma granulifera* and *Heteractis magnifica* were found to selectively target Kv1 channels. ^69^ These findings hold particular importance as Kv1 ion channels play a clinically relevant role in the pathogenesis of neurological diseases, including epilepsy. ^70^

## 4. Conclusions

Epilepsy remains one of the most critical health burdens in the world. However, the disease currently presents a significant challenge in discovering and developing therapeutic agents that are safe and effective. Venom-based therapeutics has been a subject of focus due to their potential to combat various disorders such as cardiovascular diseases and cancer. This present review demonstrates that animal venom could exert anticonvulsant effects by targeting and regulating specific receptors, modulating uptakes of molecules such as glutamate and glycine, and antagonizing ion channels. In addition, the advancement of proteomic and genomic technology has presented us with the ability to characterize and purify proteins with therapeutic potentials accurately. More proteins/compounds can be isolated and tested in-vivo for anticonvulsant activities. Animal venom represents a promising avenue for researchers to further explore and investigate its compounds as a source of potential antiepileptic/anticonvulsive agents.

## Data Availability

No data are associated with this article.
