# Supplementary material for: Animal Venoms as Potential Source of Anticonvulsants
Source: F1000Res. 2024 Mar 27;13:225. [Version 1] doi: 10.12688/f1000research.147027.1 (PMC11196940; doi:10.12688/f1000research.147027.1)
Supplement: Peer review history

# Reviewer report: Reviewer response for version 1

**DOI:** 10.5256/f1000research.161164.r293545

**Published:** 2024-07-08

**Authors:** Zhijian Cao

**Affiliations:** 1 Wuhan University, Wuhan, Hubei, China

This is a short review about Animal Venoms as Potential Source of Anticonvulsants written by Syafiq Asnawi Zainal Abidin, Anthony Kin Yip Liew, Iekhsan Othman, and Farooq Shaikh. Epilepsy is a global epidemic that affects more than 50 million people worldwide, making it one of the most common neurological diseases. Epilepsy remains one of the most critical health burdens in the world. The discovery of anti-epilepsy agents with high effect and low toxicity is still the most urgent need today and future. Animal venom is confirmed to be one significant and potential source of natural drugs including anticonvulsants. Overall, the theme of this review is very meaningful and interesting. Moreover, the structure and organization of this review is logically clear. From a scientific perspective, the article reviews and summarizes all anti-epileptic peptides and components derived from animal venom sources. Moreover, the direction and focus of future attention and research have also been proposed. All in all, I recommend that this review could be considered to be accepted after a revision. However, some minor questions still need to be modified.

**Minor points**

1. The authors should summarize and provide the molecular characteristics of the animal venom-derived anti-epileptic peptides (such as PbTx1.2.3, FrPbAII, BmK I, BmK IT2, Ppnp7, neuropolybin, Chartergellus-CP1, M-PONTX-Dq3a, M-PONTX-Dq3b, and M-PONTX, Dq3c, and so on), for example the primary amino acid sequences, cysteine pairs, molecular weights, and so on.
2. The language of this review is good. However, there are still some errors in the following. “synthesised” should be adjusted to “synthesized”; “in-vivo” should be adjusted to the italicized “in-vivo”; “mice brain” should be adjusted to “mouse brain”; “mice seizure models” should be adjusted to “mouse seizure models”.

Is the review written in accessible language?

Yes

Are all factual statements correct and adequately supported by citations?

Yes

Are the conclusions drawn appropriate in the context of the current research literature?

Yes

Is the topic of the review discussed comprehensively in the context of the current literature?

Yes

Reviewer Expertise:

Animal venom peptides

I confirm that I have read this submission and believe that I have an appropriate level of expertise to confirm that it is of an acceptable scientific standard.

# Reviewer report: Reviewer response for version 1

**DOI:** 10.5256/f1000research.161164.r293539

**Published:** 2024-06-24

**Authors:** Cletus Anes Ukwubile

**Affiliations:** 1 University of Maiduguri, Maiduguri, Borno, Nigeria

The author has rightly included in the review most essential parameters. This review draws the attention of the audience on the potentials of venom as anticonvulsant agent.

 The language use was concise and interesting. However, there is the need to provide scientific names to some of the organisms that serve as venom sources. The use of only common names are scientifically incomplete. The author should also be specific in the conclusion. For instance more proteins/compounds can be..... Proteins are compounds. It should be More compounds be...

Is the review written in accessible language?

Yes

Are all factual statements correct and adequately supported by citations?

Yes

Are the conclusions drawn appropriate in the context of the current research literature?

Yes

Is the topic of the review discussed comprehensively in the context of the current literature?

Yes

Reviewer Expertise:

Ethnopharmacology, Pharmacognosy and Natural Products Research.

I confirm that I have read this submission and believe that I have an appropriate level of expertise to confirm that it is of an acceptable scientific standard.

# Reviewer report: Reviewer response for version 1

**DOI:** 10.5256/f1000research.161164.r276839

**Published:** 2024-05-17

**Authors:** Benedict C Offor

**Affiliations:** 1 University of Johannesburg, Auckland Park, Johannesburg, South Africa

A review entitled Animal venoms as a potential source of anticonvulsants by Zainal Abidin and colleagues is very exciting and informative. Considering the challenges and stereotypes associated with the sufferers of epilepsy, there is a need to explore natural sources including venom from organisms for potential therapeutic solutions. The authors demonstrated that venom components from selected venomous organisms interact with receptors, channels, and transporters and induce anticonvulsant activities. Furthermore, venom components could stimulate GABA concentration, and modulation of L-glutamate and glycine uptake by interfering with NMDA and GABAergic transporter making them possible anticonvulsant agents.

 It would have been more interesting if the authors had presented a Figure summarising possible mechanisms of action of these venom toxins in inducing anticonvulsant activities. Thus, proposing the interactions of these venom toxins with receptors, channels, transporters and downstream responses to the perturbation.

 The review will be beneficial to both researchers in the field of venomics, pharmacology, physicians, and ordinary people. However, there are some changes I would suggest to improve the manuscript.

**Abstract**

 Add scorpion venom in the abstract since there is a sub-heading (2.2) about it and highlights about the roles in voltage-gated sodium ion channels, as a potent anticonvulsant agent.

**Introduction**

**Page 3, Paragraph 1:** Reference to back up the definition of epilepsy by the Task Force of the International League Against Epilepsy (ILAE).

**Page 3, Paragraph 2: **Ca2+, 2+ should be in superscript

**Page 3, Paragraph 3: **The first sentence could be supported with reference especially “diet changes as methods to treat epilepsy”

**Page 3, Paragraph 3:** Cl-, **- **should be in superscript

**Page 3, Paragraph 5: **I would suggest venom and venom toxins with potential effects against epilepsy since there were no approved venom-based drugs available to treat epilepsy.

**Section 2.1 sub-heading:** I suggest removing (Table 1)

**Page 4, Paragraph 1: **If “Subjects” was used to refer to “rats”, I suggest using the latter throughout

**Page 4, Paragraph 2:** “A’ of Aminobutyric should be written with lowercase font

**Table 2:** Voltage-gated?

**Table 3:** Could be moved to sub-section 2.3.

**Page 5, Paragraph 1: **in vivo?

**Page 5, Paragraph last:** *Apis mellifera* should be in italics

 The authors’ “Future directions” to explore the venoms of snakes and sea anemones is interesting, considering the role of neurotoxins (three-finger toxins) in snakebite envenoming victims. The interaction of this toxin at the neuromuscular junction inhibits presynaptic voltage-gated calcium channels by so doing causes paralysis of the snakebite victims.

Is the review written in accessible language?

Yes

Are all factual statements correct and adequately supported by citations?

Yes

Are the conclusions drawn appropriate in the context of the current research literature?

Yes

Is the topic of the review discussed comprehensively in the context of the current literature?

Yes

Reviewer Expertise:

Venomics

I confirm that I have read this submission and believe that I have an appropriate level of expertise to confirm that it is of an acceptable scientific standard.
